# Serum HMGB1 levels are independently associated with glucose clamp-derived measures of insulin resistance in women with PCOS

**DOI:** 10.1007/s40618-023-02119-y

**Published:** 2023-05-31

**Authors:** P. Moghetti, C. Catellani, C. Sartori, M. Migazzi, F. Cirillo, M. Villani, V. Buia, B. Righi, M. Dauriz, T. Fiers, F. Tosi, M. E. Street

**Affiliations:** 1 Unit of Endocrinology, Diabetes and Metabolism, Department of Medicine, University of Verona and Azienda Ospedaliera Universitaria Integrata Verona, P.le Stefani 1, 37126 Verona, Italy; 2 Department of Mother and Child, Azienda USL-IRCCS di Reggio Emilia, Reggio Emilia, Italy; 3 PhD Program in Clinical and Experimental Medicine, University of Modena and Reggio Emilia, Modena, Italy; 4 Section of Endocrinology and Diabetes, Department of Internal Medicine, South-Tyrolean Healthcare System, Bolzano General Hospital, Bolzano, Italy; 5 Laboratory for Hormonology and Department of Endocrinology, Ghent University Hospital, Ghent, Belgium; 6 Department of Medicine and Surgery, University of Parma, Parma, Italy; 7 Unit of Paediatrics, University Hospital of Parma, University of Parma, Viale A. Gramsci, 14, 43126 Parma, Italy

**Keywords:** PCOS, HMGB1, Insulin resistance, Hyperinsulinemic euglycemic clamp

## Abstract

**Purpose:**

PCOS is associated with low grade inflammation which could play a role in insulin resistance and ovarian dysfunction. Preliminary findings suggested that serum levels of HMGB1, a cytokine involved in inflammation, might be altered in women with PCOS. Primary aim of this study was to assess whether HMGB1 serum concentrations are associated with PCOS and with the state of insulin resistance of these women.

**Methods:**

Sixty women with PCOS, selected to have a similar proportion of subjects with altered or normal insulin sensitivity, and 29 healthy controls were studied. Serum HMGB1 levels were compared in subgroups of PCOS women and controls. In PCOS women, insulin sensitivity was assessed by the glucose clamp technique and HMGB1 was measured at baseline and after acute hyperinsulinemia.

**Results:**

HMGB1 levels were similar in women with PCOS and controls and no elements used for diagnosing PCOS were associated with serum HMGB1. However, HMGB1 concentrations were higher in insulin-resistant vs insulin-sensitive PCOS women (*p* = 0.017), and inversely associated with insulin-induced total and non-oxidative glucose metabolism. In both subgroups of PCOS women, serum HMBG1 levels significantly increased after acute hyperinsulinemia.

**Conclusions:**

These data suggest that HMGB1 levels are not associated with PCOS per se, but with insulin resistance. Further research should establish the underlying nature of this relationship, and whether this protein might play a role in the metabolic complications of PCOS.

## Introduction

Polycystic ovary syndrome (PCOS) is considered the most common endocrine disorder in women of reproductive age. The prevalence of this condition varies among studies, spanning from 6 to 21% even when using homogeneous diagnostic criteria [1]. PCOS is primarily characterized by hyperandrogenism, ovulatory dysfunction and polycystic ovarian morphology [2]. In addition, 70–75% of these women show insulin resistance (IR), and at least in part this appears to be independent of body fat excess [3]. However, frequency of IR may differ according to the clinical phenotypes of PCOS [4]. It has been hypothesized that hyperinsulinemia, induced by the IR status, contributes to hyperandrogenism, since insulin may enhance both ovarian and adrenal steroidogenesis [5]. Interestingly, some studies suggested that androgen excess could, in turn, hamper insulin action [6], generating a vicious circle. However, the mechanisms underlying these complex relationships in PCOS remain poorly understood.

In recent years, PCOS has been associated with systemic low-grade inflammation. Women with PCOS have been reported to have significantly higher levels of circulating inflammatory markers [7], and specific proinflammatory cytokines may interfere with insulin sensitivity [8]. Interestingly, chronic low-grade inflammation might be also a cause of ovarian dysfunction. Several factors, such as ingested macronutrients, body fat and androgens may participate in these phenomena [9].

High Mobility Group Box 1 (HMGB1) is a small protein with cytokine activity that has nuclear, cytosolic and extracellular actions. In the presence of inflammation, HMGB1 translocates from nucleus to cytosol and is actively secreted into the extracellular space, being responsible for autocrine and paracrine effects, such as activation of nuclear factor kappa light chain enhancer of activated B cells (Nf-kB) [10]. Interestingly, HMGB1 is involved with some typical IR/hyperinsulinemia-related disorders, such as type 2 diabetes and obesity [11], and its circulating concentrations have been reported to be higher in PCOS than in non-PCOS women, in a study carried out in subjects undergoing in vitro fertilization [12]. Notably, IR, inflammation and infertility are also features of cystic fibrosis (CF), a chronic inflammatory disease due to cystic fibrosis transmembrane conductance regulator (CFTR) loss of function. We previously showed that HMGB1 is increased at onset of CF-related diabetes [10]. Moreover, in vitro experiments showed that the increase in HMGB1 was dependent on CFTR malfunctioning and that insulin lowered HMGB1 [10].

The present study aimed at verifying whether HMGB1 serum concentrations are associated with both PCOS and the state of insulin resistance of these women, as assessed by the gold standard glucose clamp technique, and at verifying whether HMGB1 concentrations may acutely change during euglycemic hyperinsulinemia.

## Materials and methods

### Subjects

Sixty PCOS women, 30 subjects with reduced insulin-sensitivity (IR-PCOS) and 30 subjects with normal insulin-sensitivity (IS-PCOS)—in the age range 20–35 years, and BMI 18–30 kg/m^2^, respectively, were included in the study. Thirty normal weight healthy women were also studied, as a control group. In selection of PCOS women, insulin resistance was defined by a M-clamp value below the 25th percentile of the distribution in healthy subjects, according to the World Health Organization definition of insulin resistance [13]. All these women were recruited as part of a larger project (Verona 3P Study) aimed at building a biobank of subjects with well-characterized PCOS and healthy controls, at the University of Verona Hospital Trust.

The diagnosis of PCOS was made according to the Rotterdam criteria (presence of at least two out of three of the following criteria: clinical and/or biochemical signs of hyperandrogenism, oligo- or anovulation, and polycystic ovary morphology (PCOm); in the absence of other diseases potentially responsible for these features, such as congenital adrenal hyperplasia, thyroid disorders or hyperprolactinemia) [14]. Hyperandrogenism was defined by the presence of hirsutism (modified Ferriman and Gallwey score ≥ 8) [15], and/or by increased serum free testosterone, according to the Androgen Excess & PCOS Society consensus statement on PCOS [16] and the recommendations from the international evidence-based guideline for the assessment and management of PCOS [2]. Chronic oligo-anovulation was diagnosed by the presence of either oligomenorrhea (less than 9 menses per year) or a luteal phase serum progesterone less than 12 nmol/L. PCOm was diagnosed according to the Rotterdam workshop recommendations [14], whenever possible by using a transvaginal approach. Secondary causes were ruled out by systematic assay of serum TSH, prolactin and 17-hydroxyprogesterone, whereas other investigations were carried out when appropriate on clinical grounds. All patients received no treatments during the previous 6 months, and in particular none used oral contraceptives, insulin-sensitizing agents, anti-androgens, NSAIDs or glucocorticoids. Genetic disorders, dysmorphic features, chronic diseases, celiac disease, tumours, inflammatory diseases, or other known causes of infertility, in addition to PCOS, were additional exclusion criteria.

Control subjects had normal menstrual cycles, normal ovarian morphology, normal glucose levels, and no clinical or biochemical evidence of hyperandrogenism. After serum androgens were measured by LC–MS/MS, one control woman, with apparently normal values in preliminary routine androgen assays, showed increased serum total and free testosterone and was therefore excluded from the analysis. Hence, the control group consisted of 29 subjects.

### Protocol

All subjects underwent a complete medical examination, with measurement of body weight, height, waist and hip circumferences, and blood pressure. Hirsutism was quantified by the modified Ferriman–Gallwey score [15]. Fat mass and fat-free mass (FFM) were assessed by bioelectrical impedance (BIA 103, Akern, Florence, Italy) [17].

On day 3–8 of a spontaneous menstrual cycle or at any time, in subjects with severe menstrual dysfunction, a fasting venous blood sample was drawn for hormonal and metabolic assessment, which comprised the assay of serum gonadotropins, androgens, SHBG, total and HDL cholesterol, triglycerides and fasting plasma glucose and insulin. Moreover, in women with PCOS, plasma glucose and insulin were measured every 30 min during a 2 h oral glucose tolerance test, performed according to the WHO procedures [18].

In a separate day, insulin sensitivity was measured, in women with PCOS, by the hyperinsulinemic euglycemic clamp technique, carried out as described by DeFronzo et al. [19]. Briefly, after an overnight fasting, a continuous insulin infusion (Humulin R, Lilly, Indianapolis, IN, USA) was started and maintained at a constant rate of 80 mU/m^2^ min. Euglycemia was maintained throughout the test by a variable infusion of 20% dextrose, adjusted by monitoring plasma glucose levels in arterialized venous blood every 5–10 min. As in non-diabetic hyperandrogenic women and controls, endogenous glucose production is negligible at this insulin infusion rate [6], the amount of glucose infused into each subject can be considered equivalent to the whole-body insulin mediated glucose utilization. As muscle is responsible for most insulin-induced glucose metabolism [20], glucose disposal data (M-clamp) were expressed per fat-free mass (mg/KgFFM min). During the clamp, blood samples were collected before starting the insulin infusion and in the final steady state period of the procedure.

At baseline and during the steady state period of the clamp, indirect calorimetry was also performed over 30 min periods using a Quark RMR instrument (Cosmed, Cernusco sul Naviglio, Italy) equipped with a ventilated hood, to measure O_2_ consumption and CO_2_ production and to quantify glucose and lipid oxidation rates [21]. Non-oxidative glucose metabolism was calculated as the difference between total glucose utilization (M-clamp) and glucose oxidation during the steady state period of the clamp. Δ glucose oxidation and Δ lipid oxidation were calculated as the differences between measures obtained during the clamp and at baseline.

Only fasting endocrine and metabolic profiles were assessed in controls.

### Biochemical assays

Plasma glucose was measured by the glucose-oxidase method, using a glucose analyzer (YSI-2300 Stat Plus; YSI Inc, Yellow Springs, Ohio), and insulin was measured by an immunometric method (Biosource, Fleurus, Belgium).

HMGB1 was measured at baseline and, in subjects with PCOS only, at the end of the glucose clamp studies using a specific ELISA assay in serum (HMGB1 ELISA, Tecan, Mannendorf, Switzerland). Intra-assay and inter-assay CVs were 5.4 and 8.2%, respectively; detection limit was 0.15 ng/ml.

Measures of total testosterone and androstenedione were obtained by liquid chromatography-mass spectrometry (LC–MS/MS), using a Micromass Quattro Premier XE Mass Spectrometer from Waters Corporation (Milford, MA, USA), as previously described [22]. The free testosterone fraction (FT) was assessed by equilibrium dialysis, as previously described [22].

SHBG was assayed by an immunoradiometric method (Roche Diagnostics, Rotkreuz, Switzerland). Gonadotropins and DHEAS were measured by direct automated CLIA methods (Advia Centaur XP, Siemens, Erlangen, Germany; and Immulite 2000, Siemens).

Serum lipids were determined by standard laboratory procedures, using an automated analyzer (Dimension Vista 1500, Siemens).

### Statistical analysis

Statistical analyses were performed using STATA, version 10.1 (Stata-Corp, College Station, Texas). Qualitative variables were analysed in terms of absolute and relative frequencies. Quantitative variables were considered as mean and standard deviation.

Statistical deviation from Gaussian distribution was tested before analyses. Appropriate transformation was applied when needed to meet assumptions of normality for parametric testing, whereas transformed data were then back-transformed into the original scale, in tables and figures, for clarity. Comparisons among groups were tested using Student's *t* or chi-squared tests for continuous and dichotomic variables, respectively. For comparisons among the 3 groups, and for analysis of HMGB1 changes during the clamp in subgroups of patients, ANOVA was applied. Associations between two or more parameters were analysed using Pearson’s correlation test. Multivariable linear regression analyses were applied to identify independent predictors of selected variables. In these analyses, either serum HMGB1 or insulin sensitivity were alternatively used as the dependent variable, and clinically relevant variables such as age, fat mass, and serum FT were included as additional independent predictors. Statistical significance was considered at a two-tailed *p* < 0.05.

### Ethical committee approval

A written informed consent was subscribed by all subjects during the enrolment of an ongoing project at the University of Verona Hospital Trust (Verona 3P Study), that included the storage of serum samples in a biobank in Verona and its use for research purposes. It was conducted in accordance with the Declaration of Helsinki and approved by the institutional ethics committee. The samples were sent in dry ice to the Research Laboratory, Department of Mother and Child, Azienda USL-IRCCS of Reggio Emilia, Reggio Emilia (Italy), for HMGB1 assay; and to the Laboratory for Hormonology, Ghent University Hospital, Ghent (Belgium), for total and free testosterone and androstenedione assays. Ethical approval of this HMGB1 study was also obtained at the Azienda USL-IRCCS institution of Reggio Emilia.

## Results

### Characteristics of the study subjects

The clinical and biochemical characteristics of PCOS women and controls are reported in Table 1.

**Table 1:** Main clinical, biochemical and anthropometric characteristics of the women with PCOS and healthy controls included in the study (mean ± SD)

	PCOS	Controls	p*
Age (years)	24.1 ± 5.1	27.8 ± 3.2	**0.005**
BMI (Kg/m^2^)	26.2 ± 5.4	20.9 ± 1.9	**< 0.001**
Waist circumference (cm)	86.6 ± 13.6	72.6 ± 5.1	**< 0.001**
Fat mass (kg)	23.2 ± 10.4	12 ± 5.0	**< 0.001**
Fat-free mass (kg)	47.3 ± 4.8	43.8 ± 2.4	**0.003**
Systolic blood pressure (mmHg)	117 ± 12	110 ± 10	**0.012**
Diastolic blood pressure (mmHg)	73 ± 10	69 ± 10	0.063
Fasting glucose (mg/dL)	83.8 ± 8.5	77.5 ± 6.5	**0.012**
Fasting insulin (mU/L)	12.8 ± 10.1	6.4 ± 2.7	**0.007**
Total cholesterol (mg/dL)	161 ± 32	163 ± 19	0.752
HDL-cholesterol (mg/dL)	52.9 ± 12.4	61.7 ± 15.0	**0.010**
Triglycerides (mg/dL)	71.7 ± 45.8	62.2 ± 28.9	0.369
LH/FSH ratio	2.04 ± 1.20	0.54 ± 0.12	**0.002**
SHBG (nmol/L)	42.8 ± 20.4	70.9 ± 27.7	**< 0.001**
Total testosterone (ng/dL)	38.4 ± 14.3	28.4 ± 12.8	**0.002**
Free testosterone (ng/dL)	0.68 ± 0.29	0.32 ± 0.14	**< 0.001**
Androstenedione (ng/dL)	162 ± 56	130 ± 49	**0.010**
DHEAS (μmol/L)	5.8 ± 2.5	3.9 ± 1.2	**0.042**

*Statistically significant differences are in bold type

Anthropometric parameters, several metabolic features, systolic blood pressure, SHBG and, as expected, serum androgens differed in PCOS women with respect to healthy controls. Age differed slightly between the groups, but in the considered age range of both groups this small difference was not expected to have an effect.

The M-clamp, measured in PCOS women only, showed a mean (± SD) value in these subjects of 11.0 ± 3.3 mg/kg FFM min, which was close to the lower reference limit in our lab (11.75 mg/kg FFM min). Notably, women with PCOS were included in this study in order to have a similar proportion of subjects with normal or reduced insulin sensitivity.

The characteristics of the subgroups of PCOS women displaying insulin resistance (IR-PCOS) and insulin sensitivity (IS-PCOS) are shown in Table 2. The two subgroups differed in terms of body composition, systolic blood pressure, serum lipids, fasting insulin, glucose and insulin concentrations after the OGTT, SHBG and free testosterone, with the worse metabolic and clinical features characterizing the IR-PCOS subgroup. The M-clamp values, i.e. total glucose disposal during the glucose clamp, reflected the inclusion criteria of these subjects. However, other measures carried out during the clamp studies, i.e. insulin-induced increase of glucose oxidation and reduction of lipid oxidation, and non-oxidative metabolism of glucose during hyperinsulinemia, showed further evidence of impaired insulin action on glucose and lipid metabolism in the IR-PCOS subgroup.

**Table 2:** Main characteristics of the subgroups of insulin resistant (IR-PCOS) and insulin sensitive (IS-PCOS) women with PCOS (mean ± SD)

	IR-PCOS	IS-PCOS	p*
Age (years)	23.6 ± 5.5	24.6 ± 4.7	0.450
BMI (Kg/m^2^)	28.0 ± 5.1	24.4 ± 5.1	**0.009**
Waist circumference (cm)	91 ± 14	81 ± 11	**0.004**
Fat mass (kg)	26.1 ± 10.8	20.1 ± 9.1	**0.024**
Fat-free mass (kg)	47.8 ± 5.1	46.8 ± 4.6	0.392
Ferriman–Gallwey score	8**.**2 ± 5.5	7.5 ± 5.7	0.659
Systolic blood pressure (mmHg)	120 ± 12	114 ± 10	**0.025**
Diastolic blood pressure (mmHg)	75 ± 11	72 ± 8	0.282
Fasting glucose (mg/dL)	85 ± 10	82 ± 6	0.198
2 h OGTT glucose (mg/dL)	100 ± 34	86 ± 19	0.071
Fasting insulin (mU/L)	17.2 ± 12.0	8.1 ± 4.5	**0.001**
2 h OGTT insulin (mU/L)	100 ± 87	51 ± 29	**0.014**
Total cholesterol (mg/dL)	171 ± 35	150 ± 26	**0.012**
HDL-cholesterol (mg/dL)	49.5 ± 11.8	56.5 ± 12.2	**0.030**
Triglycerides (mg/dL)	90.5 ± 55	51.6 ± 18.9	**0.001**
LH/FSH	2.2 ± 1.4	1.9 ± 1.0	0.266
SHBG (nmol/L)	35.4 ± 17.2	50.7 ± 20.9	**0.003**
Total testosterone (ng/dL)	41.0 ± 14.6	35.6 ± 13.7	0.145
Free testosterone (ng/dL)	0.80 ± 0.30	0.55 ± 0.21	**0.001**
M-clamp (mg/kgFFM min)^a^	8.5 ± 2.4	13.6 ± 1.4	–
Δ glucose oxidation (mg/kgFFM min)	1.38 ± 1.08	2.63 ± 0.76	**< 0.001**
Nonoxidative glucose metabolism during insulin infusion (mg/kgFFM min)	5.68 ± 2.28	10.3 ± 1.68	**< 0.001**
Δ lipid oxidation (mg/kg FFM min)	0.45 ± 0.37	0.81 ± 0.35	**0.002**

*Statistically significant differences are in bold type

^a^Total insulin-induced glucose utilization

### Serum HMGB1 levels

**Fig. 1:**
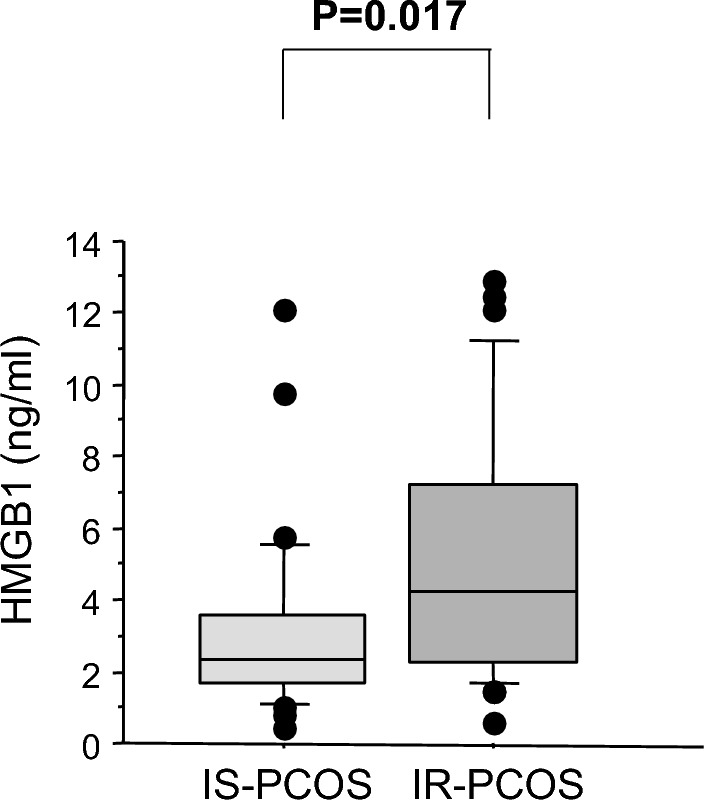
Serum HMGB1 levels in subgroups of insulin resistant and insulin sensitive women with PCOS

Serum HMGB1 levels did not differ between the whole cohort of PCOS women and controls (4.11 ± 3.22 vs 3.77 ± 2.50 ng/ml, *p* = 0.867). This finding remained unchanged after correction for age and BMI. However, HMGB1 concentrations were significantly higher in the IR-PCOS subgroup than in the IS-PCOS subgroup (Fig. 1).

Values in healthy controls were intermediate between the values measured in the two subgroups of PCOS subjects. The differences of both subgroups of PCOS patients with respect to healthy controls did not reach statistical significance.

In sensitivity analyses conducted within the whole cohort of women with PCOS, serum HMGB1 levels did not differ according to the clinical phenotypes of PCOS or the presence vs absence of each element used for this diagnosis (i.e. presence vs absence of either clinical or biochemical hyperandrogenism, altered vs preserved ovulatory capacity, or presence vs absence of PCOm) (data not shown).

In women with PCOS, serum HMGB1 was measured both at baseline conditions and during the final steady state period of the hyperinsulinemic euglycemic clamp studies, i.e. after about 2 h of exposure to increased insulin levels (mean insulin concentration in the steady state period: 209 ± 52 mU/L). Interestingly, we observed increased serum HMGB1 levels during acute hyperinsulinemia (4.77 ± 3.16 ng/ml during hyperinsulinemia vs 4.11 ± 3.22 ng/ml at baseline, *p* = 0.028). The increase was similar in the IS-PCOS and in the IR-PCOS subgroups (3.58 ± 2.61 vs 3.16 ± 2.58 ng/ml, and 5.84 ± 3.27 vs 5.00 ± 3.53 ng/ml, respectively. In ANOVA for repeated measures analysis, *p* = 0.030 for change with time, *p* = 0.833 for time-by-group interaction).

### Correlation analyses

Bivariate relationships between serum HMGB1 concentrations and clinical, biochemical, and metabolic features of the women with PCOS included in the study are reported in Table 3.

**Table 3:** Bivariate relationships between serum HMGB1 concentrations and relevant clinical features in women with PCOS

	*R*	p*
Age	− 0.275	**0.033**
BMI	0.162	0.217
Waist circumference	0.131	0.319
Fat mass	0.090	0.492
Systolic blood pressure	0.118	0.370
Diastolic blood pressure	0.034	0.796
Ferriman–Gallwey score	0.147	0.263
Fasting glucose	0.098	0.457
2 h OGTT glucose	0.266	**0.047**
Fasting insulin	0.336	**0.009**
2 h OGTT insulin	0.402	**0.005**
Total cholesterol	− 0.014	0.914
HDL-cholesterol	− 0.199	0.134
Triglycerides	0.102	0.437
LH/FSH	0.164	0.241
SHBG	− 0.187	0.152
Total testosterone	0.007	0.959
Free testosterone	0.208	0.110
M-clamp^a^	− 0.369	**0.004**
Δ glucose oxidation	− 0.197	0.201
Non-oxidative glucose metabolism during insulin infusion	− 0.539	**< 0.001**
Δ lipid oxidation	− 0.160	0.301

*Statistically significant *P* values are in bold type

^a^Total insulin-induced glucose utilization

Serum HMGB1 concentrations were directly associated with fasting insulin, and insulin and glucose levels at 2 h during the OGTT. Conversely, a statistically significant inverse association was observed between serum HMGB1 and age, whereas no relationships were found with the measured anthropometric or endocrine features.

**Fig. 2:**
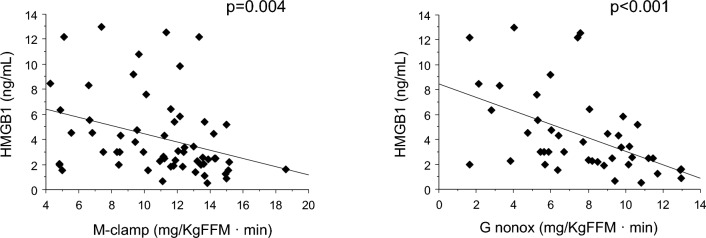
Relationships between serum HMGB1 levels and total (left panel) or non-oxidative (right panel) insulin-induced glucose metabolism

HMGB1 was inversely related to the total (M-clamp value) and the non-oxidative insulin-induced glucose metabolism (Fig. 2), whereas it was not significantly associated with the insulin-induced change of glucose or lipid oxidation.

In the multivariable analysis, serum HMGB1 levels were independently predicted, with an inverse association, by M-clamp values and age, but not by body fat mass or serum free testosterone (Table 4). Results were similar substituting M-clamp values with non-oxidative glucose metabolism (data not shown).

**Table 4:** Predictors of serum HMBG1 levels in multivariable analysis

Independent variables (explained variance 35.2%)	Standardized *β* coefficient	Standard error	*p*
Age	− 0.267	0.018	**0.030**
Fat mass	− 0.052	0.009	0.689
Free testosterone	0.072	0.363	0.600
M-clamp	− 0.344	0.033	**0.018**

Statistically significant *p* values are in bold type

Conversely, when M-clamp value was used as the dependent variable, it was independently and inversely associated with serum free testosterone, body fat mass and serum HMGB1 concentrations (Table 5).

**Table 5:** Predictors of insulin sensitivity (M-clamp) in multivariable analysis

Independent variables (explained variance 21.1%)	Standardized *β* coefficient	Standard error	*p*
Age	− 0.028	0.073	0.809
Fat mass	− 0.238	0.035	**0.038**
Free testosterone	− 0.350	1.306	**0.004**
HMGB1	− 0.282	0.497	**0.018**

Statistically significant *p* values are in bold type

## Discussion

This study compared serum HMGB1 levels in a cohort of women with PCOS, selected in order to have a similar proportion of subjects with altered or normal insulin sensitivity, and healthy controls. We observed that HMGB1 levels were higher in insulin-resistant PCOS women as compared with insulin-sensitive PCOS women, whereas levels in either the whole group or in both these subgroups of PCOS women did not significantly differ from those in healthy controls. None of the clinical elements used for the diagnosis of PCOS, based on the Rotterdam criteria (i.e. hyperandrogenism, oligo-anovulation and polycystic ovarian morphology), were associated with HMGB1 levels. Furthermore, serum HMGB1 did not show any relationship with the anthropometric features. However, in women with PCOS, serum HMGB1 was directly associated with fasting serum insulin and glucose and insulin response to oral glucose. In addition, serum HMGB1 was inversely associated with both total glucose disposal (M-clamp value) and non-oxidative glucose metabolism during hyperinsulinemia. Finally, in these PCOS women, serum HMBG1 levels slightly but significantly increased after short term exposure to marked hyperinsulinemia, induced by the glucose clamp procedure, with similar behaviors in insulin sensitive and insulin resistant subgroups of patients.

A few previous studies investigated serum HMGB1 levels in women with PCOS, reporting increased levels in these subjects, as compared to healthy controls [12, 23, 24]. Similar findings were reported by one of these studies in the follicular fluid of women with PCOS submitted to in vitro fertilization, as compared to non-PCOS subjects undergoing the same procedure [12]. In these studies, serum HMGB1 levels directly correlated with plasma insulin levels, suggesting an association with insulin resistance. Consistently, it was reported that serum HMGB1 levels were lowered in women with PCOS after therapy with the insulin sensitizer metformin [23], or with the putative mediator of insulin signaling myo-inositol, combined with alpha-lipoic acid [24]. Nevertheless, in contrast with findings in serum, HMGB1 concentrations were inversely associated with insulin levels in the follicular fluid of women with PCOS submitted to IVF [12]. This result is not easily explained. A dysregulation of insulin internalization and transport to the ovarian follicular fluid could be hypothesized in insulin-resistant women with PCOS.

Two previous studies, carried out in subjects with cystic fibrosis-related diabetes and in a lipopolysaccharide-induced septic rat model, respectively, have shown that HMGB1 was increased in the presence of hyperglycaemia and was lowered after insulin therapy [10, 25]. This suggested that insulin might acutely reduce HMGB1 levels. Conversely, in our study serum HMGB1 increased during the clamp studies, after short-term tissue exposure to markedly increased insulin levels. These discrepancies may possibly be related to differences in duration and/or extent of hyperinsulinemia. An alternative hypothesis, which appears more likely if we hypothesize that hyperglycemia is a factor responsible for HMGB1 increase, is that the results obtained in previous studies were due to the insulin-induced attenuation of hyperglycemia rather than to a direct effect of insulin on HMGB1 levels.

Nevertheless, a reduction of HMGB1 expression was also found in an epithelial bronchial cell model for cystic fibrosis, after in vitro insulin exposure [10]. Available information is insufficient to understand whether this could be due to inflammation being a stronger driver on HMGB1 levels in cystic fibrosis or if there may be a different regulation in PCOS.

In our study, HMGB1 levels were inversely associated with both total and non-oxidative metabolism of glucose, but not with insulin-induced stimulation of glucose oxidation or suppression of lipid oxidation. These findings may suggest a specific link between HMGB1 levels and the non-oxidative pathway of glucose metabolism, which is mainly represented by glycogen synthesis and is responsible for the largest part of glucose metabolism in our experimental conditions (i.e. the hyperinsulinemic euglycemic clamp at a relatively high insulin infusion rate). Interestingly, glycogen synthase kinase-3β (GSK-3β) is a serine threonine kinase that can phosphorylate a number of different proteins in a variety of different pathways. Its regulatory effects include an enhancement of the NF-κB-dependent inflammatory pathway and a decrease of glycogen synthase activity in the liver and muscle [26, 27]. Therefore, a role of GSK-3β activation in this relationship might be hypothesized, although the mechanisms potentially involved remain to be understood.

The present data suggest that serum HMGB1 is unaffected by the PCOS status per se, and it can be hypothesized that previous findings in the comparison between PCOS women and healthy controls may indeed reflect the differences in the expected frequency of insulin resistance between the two groups. Our previous studies, carried out using the glucose clamp technique, showed that this frequency may be around 75% in women with PCOS (3). Conversely, in the present study our cohort of PCOS women was enriched of insulin sensitive subjects, due to the predefined inclusion criteria of an identical proportion of subjects with altered or normal insulin sensitivity. In this regard, the intermediate values measured in our healthy women, as compared to the insulin resistant and insulin sensitive subgroups of PCOS women, may also derive from the selection criteria used in our protocol. In actual facts, according to the WHO criteria [13] we used in determining the cut-off for the presence or the absence of insulin resistance in PCOS women, the limit for diagnosis of insulin resistance corresponds to the lower quartile of values in the reference population. Thus, by definition our control group may possibly contain a 25% proportion of insulin resistant individuals, whereas our subgroups of PCOS women consisted entirely of either insulin resistant or insulin-sensitive individuals. However, this hypothesis is merely speculative, as insulin sensitivity was not measured in controls.

In our study, in the multivariable analysis, serum HMGB1 was independently predicted by insulin sensitivity, but not by fat mass or serum androgens, further supporting an association between HMGB1 and insulin action. The design of our study was cross-sectional and thus it might be affected by reverse causation as we cannot establish the direction of this relationship. However, we have observed that serum HMGB1 levels significantly increased during insulin infusion in clamp studies, when high levels of insulin (around 200 mU/L) were maintained for about 2 h. These insulin concentrations can powerfully stimulate insulin receptors in peripheral tissues. Moreover, it was reported that exposure to high insulin concentrations induced apoptosis of cultured rat granulosa cells and this phenomenon was accompanied by increased extracellular HMGB1 concentrations [28]. These findings are consistent with the hypothesis of increased insulin as the *primum movens* in this association. Nevertheless, recent data showed that increased HMGB1 may impair insulin signaling in granulosa cells obtained from PCOS patients [29], suggesting that both directions in the relationship between HMGB1 and insulin resistance are potentially working. We can thus hypothesize that HMGB1 might participate in one of the numerous vicious circles underlying the pathophysiology of insulin resistance in PCOS.

At variance with our findings, Wang et al. previously reported a statistically significant direct relationship between HMGB1 concentrations and serum testosterone, as measured by a chemiluminescent method [23]. This assay is inaccurate for measuring serum androgens in women [22] and the methodological differences could account for the different findings between studies. Moreover, a relationship between serum testosterone and insulin resistance has been consistently reported in women with PCOS [30], and it could be responsible for the results reported in the Wang study. Unfortunately, these authors did not use a multivariable analysis to verify whether the association between serum testosterone and HMGB1 levels was independent of insulin resistance.

Strengths of our study are the gold standard procedures used for measuring insulin sensitivity and serum androgens. Limitations to be considered are the cross-sectional experimental design, which precludes the possibility of establishing clear cause-effect relationships, and the assay of total HMGB1, which includes fractions with different redox forms and potentially different biological characteristics. Additional potential limitations are the sample size of the control group, which was relatively small, and the difference in BMI between groups. However, the latter does not appear a factor precluding the comparison between groups of HMGB1 levels, as BMI did not show any relationship with HMGB1 concentrations in our cohort, and correction of data for this variable did not affect the results. Nevertheless, we acknowledge that comparison of HMGB1 levels between women with PCOS and healthy controls may require further research.

In conclusion, these data suggest that serum HMGB1 levels are not associated with PCOS or with the more typical features of this syndrome. However, in women with PCOS, HMGB1 levels show an independent association with insulin resistance, and increase during short-term hyperinsulinemia. Further research should establish the direction of this relationship and clarify whether this protein may play a role in the pathogenesis and/or in the metabolic complications of PCOS.

## Data Availability

Some or all datasets generated during and/or analyzed during the current study are not publicly available but are available from the corresponding author on reasonable request.

## References

[1] Lizneva D, Suturina L, Walker W, Brakta S, Gavrilova-Jordan L, Azziz R (2016). Criteria, prevalence, and phenotypes of polycystic ovary syndrome. Fertil Steril.

[2] Teede HJ, Misso ML, Costello MF, Dokras A, Laven J, Moran L, Piltonen T, Norman RJ, International PCOS Network (2018). Recommendations from the international evidence-based guideline for the assessment and management of polycystic ovary syndrome. Hum Reprod.

[3] Tosi F, Bonora E, Moghetti P (2017). Insulin resistance in a large cohort of women with polycystic ovary syndrome: a comparison between euglycaemic-hyperinsulinaemic clamp and surrogate indexes. Hum Reprod.

[4] Moghetti P, Tosi F, Bonin C, Di Sarra D, Fiers T, Kaufman JM, Giagulli VA, Signori C, Zambotti F, Dall’Alda M, Spiazzi G, Zanolin ME, Bonora E (2013). Divergences in insulin resistance between the different phenotypes of the polycystic ovary syndrome. J Clin Endocrinol Metab.

[5] Moghetti P, Tosi F (2021). Insulin resistance and PCOS: chicken or egg?. J Endocrinol Invest.

[6] Moghetti P, Tosi F, Castello R, Magnani CM, Negri C, Brun E, Furlani L, Caputo M, Muggeo M (1996). The insulin resistance in women with hyperandrogenism is partially reversed by antiandrogen treatment: evidence that androgens impair insulin action in women. J Clin Endocrinol Metab.

[7] Escobar-Morreale HF, Luque-Ramírez M, González F (2011). Circulating inflammatory markers in polycystic ovary syndrome: a systematic review and metaanalysis. Fertil Steril.

[8] González F, Considine RV, Abdelhadi OA, Acton AJ (2020). Inflammation triggered by saturated fat ingestion is linked to insulin resistance and hyperandrogenism in polycystic ovary syndrome. J Clin Endocrinol Metab.

[9] Moncayo S, Insenser M, Martínez-García MÁ, Fuertes-Martín R, Amigó-Grau N, Álvarez-Blasco F, Luque-Ramírez M, Correig-Blanchar X, Escobar-Morreale HF (2021). Acute-phase glycoprotein profile responses to different oral macronutrient challenges: Influence of sex, functional hyperandrogenism and obesity. Clin Nutr.

[10] Montanini L, Cirillo F, Smerieri A, Pisi G, Giardino I, d'Apolito M, Spaggiari C, Bernasconi S, Amarri S, Street ME (2016). HMGB1 is increased by CFTR loss of function, is lowered by insulin, and increases in vivo at onset of CFRD. J Clin Endocrinol Metab.

[11] Wang Y, Zhong J, Zhang X, Liu Z, Yang Y, Gong Q, Ren B (2016). The role of HMGB1 in the pathogenesis of type 2 diabetes. J Diabetes Res.

[12] Cirillo F, Catellani C, Sartori C, Lazzeroni P, Morini D, Nicoli A, Giorgi-Rossi P, Amarri S, La Sala GB, Street ME (2019). CFTR and FOXO1 gene expression are reduced and HMGB1 is increased in the ovaries and serum of women with Polycystic Ovarian Syndrome. Gynecol Endocrinol.

[13] World Health Organization (1999). Definition, diagnosis and classification of diabetes mellitus and its complications. Report of a WHO consultation. Part 1: diagnosis and classification of diabetes mellitus.

[14] Rotterdam ESHRE/ASRM-Sponsored PCOS Consensus Workshop Group (2004). Revised 2003 consensus on diagnostic criteria and long term health risks related to polycystic ovary syndrome (PCOS). The Rotterdam ESHRE/ASRM-sponsored PCOS consensus workshop group. Hum Reprod.

[15] Escobar-Morreale HF, Carmina E, Dewailly D, Gambineri A, Kelestimur F, Moghetti P, Pugeat M, Qiao J, Wijeyaratne CN, Witchel SF, Norman RJ (2012). Epidemiology, diagnosis and management of hirsutism: a consensus statement by the Androgen Excess and Polycystic Ovary Syndrome Society. Hum Reprod Update.

[16] Azziz R, Carmina E, Dewailly D, Diamanti-Kandarakis E, Escobar-Morreale HF, Futterweit W, Janssen OE, Lergo RS, Norman RJ, Taylor AE, Witchel SF (2009). The Androgen Excess and PCOS Society criteria for the polycystic ovary syndrome: the complete task force report. Fertil Steril.

[17] Gray DS, Bray GA, Gemayel N, Kaplan K (1989). Effect of obesity on bioelectrical impedance. Am J Clin Nutr.

[18] World Health Organization Study Group (1985). Diabetes mellitus. World Health Organ Tech Rep Ser.

[19] DeFronzo RA, Tobin JD, Andres R (1979). Glucose clamp technique: a method for quantifying insulin secretion and resistance. Am J Physiol.

[20] DeFronzo RA (1988). The triumvirate: beta-cell, muscle, liver. A collusion responsible for NIDDM. Diabetes.

[21] Ferrannini E (1988). The theoretical bases of indirect calorimetry: a review. Metabolism.

[22] Tosi F, Fiers T, Kaufman JM, Dall'Alda M, Moretta R, Giagulli VA, Bonora E, Moghetti P (2016). Implications of androgen assay accuracy in the phenotyping of women with polycystic ovary syndrome. J Clin Endocrinol Metab.

[23] Wang H-H, Lin M, Xiang G-D (2018). Serum HMGB1 levels and its associations with endothelial dysfunction in patients with PCOS. Physiol Res.

[24] Cirillo F, Catellani C, Lazzeroni P, Sartori C, Tridenti G, Vezzani C, Fulghesu AM, Madeddu E, Amarri S, Street ME (2020). HMGB1 is increased in adolescents with polycystic ovary syndrome (PCOS) and decreases after treatment with myo-inositol (MYO) in combination with alpha-lipoic acid (ALA). Gynecol Endocrinol.

[25] Hagiwara S, Iwasaka H, Hasegawa A, Koga H, Noguchi T (2008). Effects of hyperglycemia and insulin therapy on high mobility group box 1 in endotoxin-induced acute lung injury in a rat model. Crit Care Med.

[26] Cortés-Vieyra R, Bravo-Patiño A, Valdez-Alarcón JJ, Juárez MC, Finlay BB, Baizabal-Aguirre VM (2012). Role of glycogen synthase kinase-3β in the inflammatory response caused by bacterial pathogens. J Inflamm (Lond).

[27] Beurel E, Grieco SF, Jope RS (2015). Glycogen synthase kinase-3 (GSK3): regulation, actions, and diseases. Pharmacol Ther.

[28] Ni XR, Sun ZJ, Hu GH, Wang RH (2015). High concentration of insulin promotes apoptosis of primary cultured rat ovarian granulosa cells via its increase in extracellular HMGB1. Reprod Sci.

[29] Zhang C, Hu J, Wang W, Sun Y, Sun K (2020). HMGB1-induced aberrant autophagy contributes to insulin resistance in granulosa cells in PCOS. FASEB J.

[30] Tosi F, Di Sarra D, Kaufman JM, Bonin C, Moretta R, Bonora E, Zanolin E, Moghetti P (2015). Total body fat and central fat mass independently predict insulin resistance but not hyperandrogenemia in women with polycystic ovary syndrome. J Clin Endocrinol Metab.

